# BrTCP7 Transcription Factor Is Associated with MeJA-Promoted Leaf Senescence by Activating the Expression of *BrOPR3* and *BrRCCR*

**DOI:** 10.3390/ijms20163963

**Published:** 2019-08-14

**Authors:** Yan-mei Xu, Xian-mei Xiao, Ze-xiang Zeng, Xiao-li Tan, Zong-li Liu, Jian-wen Chen, Xin-guo Su, Jian-ye Chen

**Affiliations:** 1 State Key Laboratory for Conservation and Utilization of Subtropical Agro-bioresources/Guangdong Provincial Key Laboratory of Postharvest Science of Fruits and Vegetables/Engineering Research Center of Southern Horticultural Products Preservation, Ministry of Education, College of Horticulture, South China Agricultural University, Guangzhou 510642, China; 2 Scientific Observing and Experimental Station of Crop Cultivation in South China, Ministry of Agriculture, College of Agriculture, South China Agricultural University, Guangzhou 510642, China; 3 Department of Food Science, Guangdong Food and Drug Vocational College, Guangzhou 510520, China

**Keywords:** Chinese flowering cabbage, leaf senescence, JA, transcriptional activation

## Abstract

The plant hormone jasmonic acid (JA) has been recognized as an important promoter of leaf senescence in plants. However, upstream transcription factors (TFs) that control JA biosynthesis during JA-promoted leaf senescence remain unknown. In this study, we report the possible involvement of a TEOSINTE BRANCHED1/CYCLOIDEA/PCF (TCP) TF BrTCP7 in methyl jasmonate (MeJA)-promoted leaf senescence in Chinese flowering cabbage. Exogenous MeJA treatment reduced maximum quantum yield (Fv/Fm) and total chlorophyll content, accompanied by the increased expression of senescence marker and chlorophyll catabolic genes, and accelerated leaf senescence. To further understand the transcriptional regulation of MeJA-promoted leaf senescence, a class I member of TCP TFs BrTCP7 was examined. BrTCP7 is a nuclear protein and possesses trans-activation ability through subcellular localization and transcriptional activity assays. A higher level of *BrTCP7* transcript was detected in senescing leaves, and its expression was up-regulated by MeJA. The electrophoretic mobility shift assay and transient expression assay showed that BrTCP7 binds to the promoter regions of a JA biosynthetic gene *BrOPR3* encoding OPDA reductase3 (OPR3) and a chlorophyll catabolic gene *BrRCCR* encoding red chlorophyll catabolite reductase (RCCR), activating their transcriptions. Taken together, these findings reveal that BrTCP7 is associated with MeJA-promoted leaf senescence at least partly by activating JA biosynthesis and chlorophyll catabolism, thus expanding our knowledge of the transcriptional mechanism of JA-mediated leaf senescence.

## 1. Introduction

As one of the major leafy vegetables, Chinese flowering cabbage (*Brassica rapa* ssp. *Parachinensis*) is a popular in the Asian diet due to its health-promoting compounds and valuable anticancer properties [1,2,3]. Generally, flowering shoots, stems, and younger leaves of this cabbage are harvested for eating. However, harvested cabbage leaves senesce rapidly during transportation and storage, wilt, and yellow, reducing product quality and commercial value [4,5,6]. Thus, there is a demand for the elucidation of the molecular mechanisms of leaf senescence of Chinese flowering cabbage. Such knowledge is important for developing practical solutions to maintain the quality and extend the shelf-life of this vegetable.

Previous genetic and molecular studies have demonstrated that leaf senescence is an active biological process that is tightly regulated by various intrinsic and external factors, such as developmental stage, natural plant hormones, and stresses [7,8,9,10,11]. Among the various phytohormones, jasmonic acid (JA) and its derivate methyl jasmonate (MeJA), have been reported to accelerate leaf senescence [12,13,14]. In a variety of plants such as Arabidopsis, rice, and maize, endogenous JA is found to be significantly accumulated during leaf senescence [15,16,17]. Exogenous JA/MeJA application can accelerate leaf yellowing and senescence and enhance the expression of several senescence-associated genes (*SAGs*) including chlorophyll catabolic genes (*CCGs*), as well as genes involved in the JA biosynthesis and signaling pathways [18].

Evidence suggests that transcription factors (TFs) are important regulatory proteins that control the onset and progression of JA-induced leaf senescence by altering the expression of *SAGs* [11,14]. For example, Arabidopsis MYC2, 3, and 4 function redundantly to activate JA-induced leaf senescence by directly activating the expression of *SAG29* and *CCGs* [18]. In contrast, bHLH subgroup IIId TFs bHLH03, 13, 14, and 1, target the *SAG29* promoter and repress its expression [19]. WRKY57 directly suppresses the transcription of *SEN4* and *SAG12*, acting as a negative regulator of JA-induced leaf senescence in Arabidopsis [20]. These findings highlight the complicated regulatory network of JA-promoted leaf senescence governed by a series of TFs, promoting the identification of other TFs associated with this process.

TEOSINTE BRANCHED1/CYCLOIDEA/PCF (TCP) proteins, with the conserved TCP domains, are plant-specific TFs that have been identified in many plant species including Arabidopsis [21], rice [22], maize [23], tomato [24], and Chinese cabbage [25]. TCP TFs have been well documented to participate in multiple biological processes such as cell proliferation and growth, and stress response [26]. Several TCP TFs have been shown to mediate hormone-induced changes in cell proliferation and act as modulators, or even as key players of hormone synthesis, transport, and signal transduction [26]. For instance, Arabidopsis TCP15 is required for the optimal balance between auxin levels and cytokinins responses in the developing carpel [27]. Rice OsTCP19 is strongly associated with abscisic acid (ABA)-mediated abiotic stress responses by binding and modulating the activity of *OsABI4* (ABSCISIC ACID INSENSITIVE4), and overexpression of OsTCP19 in Arabidopsis affects not only ABA but also auxin and JA signaling [28]. GhTCP19 from gladiolus was shown to be a positive regulator of the corm dormancy release process by repressing the expression of an ABA biosynthesis gene expression (*GhNCED*, 9-cis-epoxycarotenoid dioxygenase), as well as promoting cytokinins biosynthesis (*GhIPT*, isopentenyl transferase) and signal transduction (*GhARR*, ARABIDOPSIS RESPONSE REGULATORS) [29]. Genome-wide transcriptional analyses revealed that JA-related global responses are affected by gain or loss of TCP activity [30]. Breeze et al. conducted transcriptome analysis and suggested the role of TCPs during leaf senescence in Arabidopsis [31]. Three Arabidopsis TCP TFs, TCP20, TCP9, and TCP4, were further found to target the promoter of a JA biosynthetic gene *LOX2* (Lipoxygenase2), repressing and activating its transcription, thus acting antagonistically controlling JA-mediated leaf development and senescence [32]. The involvement of TCP members in JA-promoted leaf senescence as well as the associated regulatory mechanism remain to be elucidated.

Previously, we identified and characterized several TFs including BrWRKY65 [1], BrWRKY6 [2], BrERF72 [5], and BrNAC055 [6] involved in leaf senescence in Chinese flowering cabbage, of which BrERF72 was shown to enhance JA accumulation by inducing the expression of three JA biosynthetic genes (*BrLOX4*, *BrAOC3*, and *BrOPR3*) during MeJA-promoted leaf senescence [5]. Leaf senescence is a complex biological process mediated by numerous TFs, and the underpinning regulatory mechanisms vary from species to species and with growing conditions. The current study revealed that a TCP, TF BrTCP7, is positively associated with MeJA-promoted leaf senescence by the direct transcriptional activation of a JA biosynthetic gene *BrOPR3* and a *CCG BrRCCR*.

## 2. Results and Discussion

### 2.1. Leaf Senescence of Chinese Flowering Cabbage is Promoted by Exogenous MeJA Treatment

**Figure 1:**
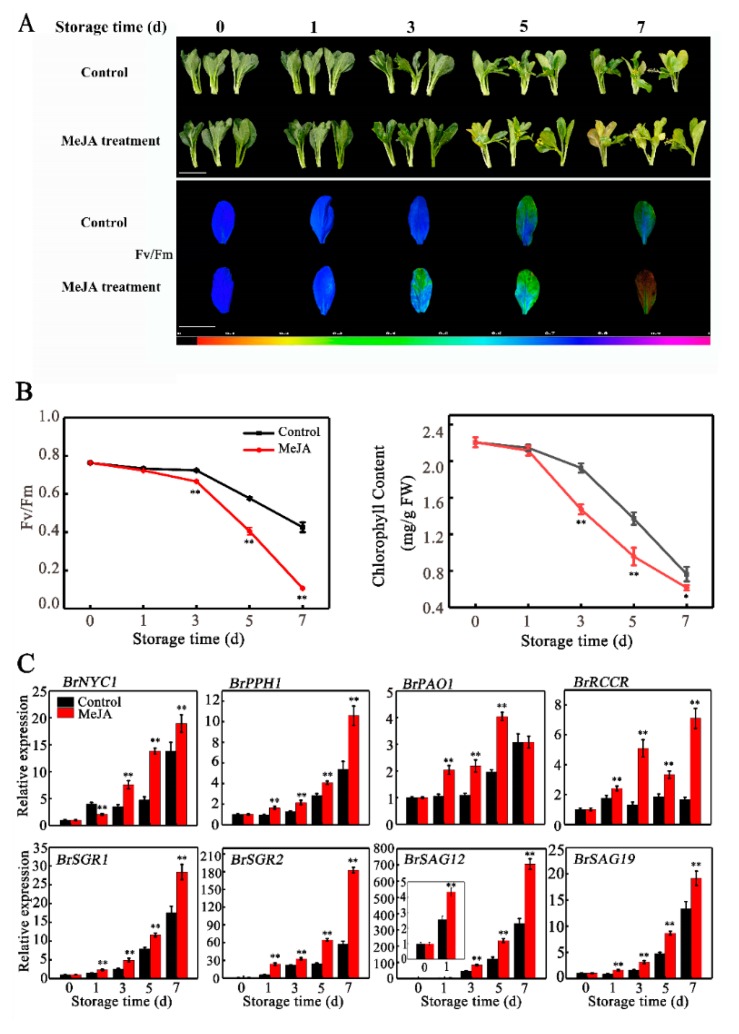
Methyl jasmonate (MeJA) treatment promotes leaf senescence of Chinese flowering cabbage. (**A**) Appearance and chlorophyll fluorescence imaging (Fv/Fm) of control and MeJA-treated cabbage leaves during senescence. The false color code depicted at the bottom of the image ranges from 0 (black) to 1.0 (purple). Bar = 10 cm. (**B**) Changes in Fv/Fm and total chlorophyll content in control and MeJA-treated cabbage leaves during senescence. (**C**) Relative expression of six *CCGs* (*BrNYC1*, *BrPPH1*, *BrPAO1*, *BrRCCR*, *BrSGR1* and *BrSGR2*) and two senescence-marker genes (*BrSAG12* and *BrSAG19*) in control and MeJA-treated cabbage leaves during senescence. Data presented in (**B**) and (**C**) are the mean ± SD of three biological replicates. Asterisks indicate a significant difference in MeJA-treated leaves compared with control leaves (Student’s *t*-test: * *p* < 0.05 and ** *p* < 0.01).

The phytohormone JA-induced leaf senescence has been observed in several plant species [14,15,16,17,18,19]. Our previous study showed that exogenous MeJA treatment promotes leaf senescence of Chinese flowering cabbage [5], which was further verified in the present work. As shown in Figure 1A, appearance and non-invasive chlorophyll fluorescence imaging with maximum quantum yield (*Fv*/*Fm*) default demonstrated that, in comparison to the control, MeJA-treated cabbage leaves exhibited a greater degree of yellowing on the fifth and seventh day of storage. As expected, the *Fv*/*Fm* value and total chlorophyll content were significantly lower in MeJA-treated leaves, which were 70.7% and 70.1% of the control leaves, respectively, on the fifth day of storage (Figure 1B). To investigate why MeJA promotes leaf senescence, we quantified the relative abundance of transcripts of two *SAGs* (*BrSAG12* and *BrSAG19*) and six *CCGs* (*BrPAO*, *BrPPH*, *BrNYC1*, *BrRCCR*, *BrSGR1,* and *BrSGR2*) after MeJA treatment. The transcript levels of all these genes increased more dynamically upon treatment with MeJA, with a 2.89-, 1.44-, 2.03-, 1.81-, 1.47-, 2.62-, 1.93-, and 1.83-fold induction for *BrNYC1*, *BrPPH*, *BrPAO*, *BrRCCR*, *BrSGR1*, *BrSGR2*, *BrSAG12*, and *BrSAG19*, respectively on the fifth day of storage compared with the control (Figure 1C).

### 2.2. BrTCP7 is A Member of Class I TCP

Transcriptional regulation mediated by TFs, such as MYCs, NACs, and WRKYs, plays an important role in JA-induced leaf senescence [14,33]. Numerous TCP TFs have been identified in plants; however, only few of them were reported to be involved in JA-mediated leaf development and senescence [26,32]. Thus, we focused on the identification of TCP TFs from our RNA-seq transcriptome database associated with Chinese flowering cabbage leaf senescence. Using a false discovery rate (FDR) of less than 0.05 and a fold change larger than 0.5 as thresholds, six genes annotated as TCP proteins were found to be up-regulated (unpublished data). Among these *TCPs*, the member (GenBank number, XM_009152613.2) that was most up-regulated during leaf senescence, was selected for further investigation.

**Figure 2:**
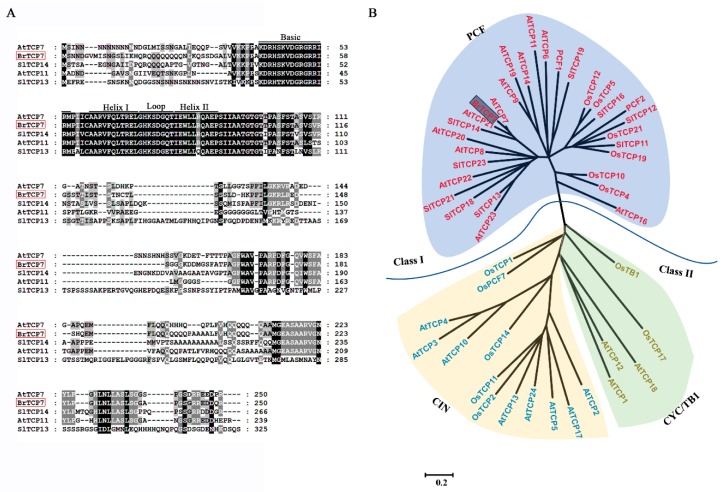
Multiple sequence alignment and phylogenetic analysis of BrTCP7. (**A**) Multiple alignment of BrTCP7 with other TCP members. The following proteins were used for analysis: AtTCP7 (NP_197719.1), AtTCP11 (NP_196450.1), SlTCP13 (NP_001233966.1), and SlTCP14 (NP_001293140.1). Identical and similar amino acids are shaded in black and grey, respectively. Single underlining indicates the conserved non-canonical basic helix–loop–helix structure. (**B**) Phylogenetic analysis of TCPs. BrTCP7 is boxed. The phylogenetic tree was constructed using the maximum likelihood inference method using the MEGA program (version 5.0,Institute of Molecular Evolutionary Genetics, The Pennsylvania State University, PA, USA).

The resulting amplified full-length gene was 753 bp, encoding a protein with 250 amino acid residues, with a calculated molecular weight of 26.97 kDa, and a *p*I value of 9.69. By blasting the NCBI database, we found that this gene shares high similarity (74.9%) with Arabidopsis AtTCP7, and thus was named BrTCP7. The most distinguished characteristic of TCP proteins is the presence of a ~59-amino-acid-long conserved region with a non-canonical basic helix-loop-helix (bHLH) structure (the TCP domain) in their N-terminal, which was proven to be responsible for nuclear targeting, DNA binding, and protein–protein interactions [25,34]. Multiple-alignment of BrTCP7 showed that, similar to other orthologous TCP proteins, BrTCP7 contains the conserved TCP domain (Figure 2A). On the basis of the TCP domain, the TCP members can be grouped into two distinct subfamilies: class I (PCF or TCP-P class) and class II (TCP-C class), among which class II can be further divided into subclades CIN and CYC/TB1 [26,35]. As shown in Figure 2B, the phylogenetic tree shows that BrTCP7 together with AtTCP7, AtTCP14, and SlTCP14 belong to class I.

### 2.3. Molecular Properties of BrTCP7

**Figure 3:**
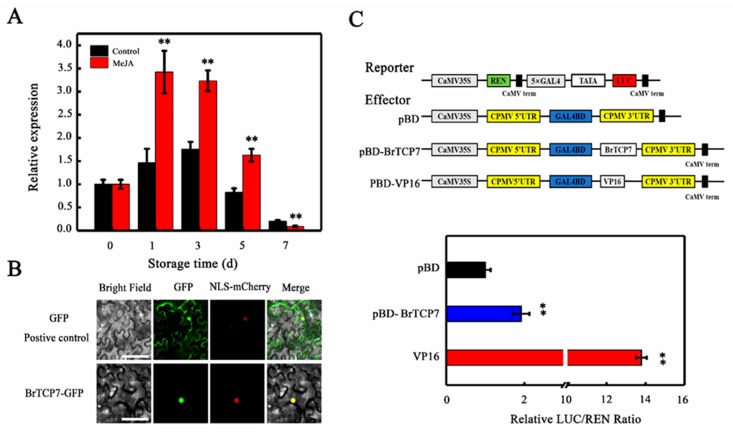
Molecular properties of BrTCP7. (**A**) Relative expression of BrTCP7 in control and MeJA-treated cabbage leaves during senescence. Each value represents the mean ± SD of three biological replicates. Asterisks indicate a significant difference in MeJA-treated leaves compared with control leaves (Student’s *t*-test: * *p* < 0.05 and ** *p* < 0.01). (**B**) Subcellular localization of BrTCP7 in epidermal cells of *Nicotiana benthamiana* leaves. A plasmid harboring GFP or BrTCP7-GFP was transformed into *N. benthamiana* leaves by *Agrobacterium tumefaciens* strain EHA105. GFP signals were observed with a fluorescence microscope after two days of infiltration. Bars = 30 μm. (**C**) Trans-activation of BrTCP7 in *N. benthamiana* leaves. The trans-activation ability of BrTCP7 was indicated by the ratio of luciferase (LUC) to renilla luciferase (REN). The LUC/REN ratio of the empty pBD vector (negative control) was used as a calibrator (set to 1). pBD-VP16 was used as a positive control. Data are means ± SD of six independent biological replicates. Asterisks represent significant differences at the 0.01 level by Student’s *t*-test compared to pBD.

To evaluate whether *BrTCP7* is associated with MeJA-induced leaf senescence, we quantified its expression in Chinese flowering cabbage during leaf senescence. qRT-PCR analysis showed that, consistent with the RNA-seq data, the transcript level of *BrTCP7* increases during leaf senescence. More pronounced elevation of the *BrTCP7* transcript was observed in MeJA-treated leaves, which were ~0.85- and 0.51-fold higher than that of the control leaves on days 3 and 5 of storage, respectively (Figure 3A). Generally, TCP proteins are localized in nuclei [34,36,37]. Subcellular localization prediction also indicated that BrTCP7 is located in the nuclear region. To verify this prediction, *BrTCP7* was fused to green fluorescent protein (GFP) and transiently expressed in *Nicotiana benthamiana* leaves. Compared with the fluorescence of control 35S-GFP that is distributed throughout the cell, BrTCP7-GFP fusion protein, like the nuclear marker (NLS-mCherry), was exclusively detected in the nucleus (Figure 3B). To provide evidence for potential roles of BrTCP7 in transcriptional regulation, we assessed the transcriptional activity of BrTCP7 in vivo using the dual-luciferase reporter system. As shown in Figure 3C, similar with the activator control VP16, co-infiltration of *BrTCP7* with the reporter significantly increased the luciferase (LUC)/renilla luciferase (REN) ratio. These data reveal that BrTCP7 is a nuclear-localized transcriptional activator that is possibly associated with MeJA-induced leaf senescence of Chinese flowering cabbage.

### 2.4. BrTCP7 Directly Binds to the Promoter of BrOPR3 and BrRCCR

**Figure 4:**
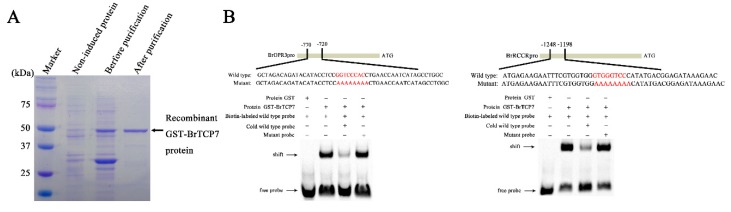
BrTCP7 directly binds to *BrOPR3* and *BrRCCR* promoters. (**A**) SDS-PAGE gel stained with Coomassie brilliant blue demonstrating affinity purification of the recombinant glutathione-S-transferases (GST)-tagged BrTCP7 protein used for EMSA. Recombinant GST-BrTCP7 protein is indicated by the arrow. (**B**) EMSA showing the binding of BrTCP7 to the TCP binding site of the *BrOPR3* or *BrRCCR* promoter. Purified GST-BrTCP7 protein was incubated with the biotin-labeled wild-type probe containing the TCP binding site, and the DNA–protein complexes were separated on native polyacrylamide gels. Sequences of both the wild-type and mutant probes are shown at the top of the image (wild-type and mutant TCP binding site are marked with red letters). The mutant probe was used to test binding specificity. Shifted bands, suggesting the formation of DNA–protein complexes, are indicated by arrows. − represents absence, + represents presence. Competition experiments were conducted by adding 250-fold molar excess of cold probe (unlabeled wild-type DNA fragment) or mutant probe.

We then intended to reveal the regulatory mechanism of BrTCP7 involved in MeJA-induced leaf senescence of Chinese flowering cabbage. For this purpose, it is vital to identify the potential targets of BrTCP7. Molecular analyses showed that the predicted consensus binding site of class I TCP TFs is GGNCCCAC, especially the core sequence GCCCR [26,32,34]. After surveying the promoter sequences of three JA biosynthetic genes (*BrLOX4*, *BrAOC3*, and *BrOPR3*) as well as six *CCGs* (*BrPAO*, *BrPPH*, *BrNYC1*, *BrRCCR*, *BrSGR1,* and *BrSGR2*) that were previously reported to be associated with leaf senescence in Chinese flowering cabbage [2,5,38], the TCP binding site was found in *BrOPR3* and *BrRCCR* promoters (Text S1). To confirm the binding of BrTCP7 to *BrOPR3* and *BrRCCR* promoters, an in vitro electrophoretic mobility shift assay (EMSA) was performed. Results showed that purified GST-BrTCP7 protein (Figure 4A), not the GST protein, binds to the TCP binding elements presented in *BrOPR3* and *BrRCCR* promoters, causing a mobility shift band (Figure 4B). Adding excess amount (250-fold) of the cold probe (unlabeled wild-type DNA fragment) but not the mutant probe eliminated the binding complex (Figure 4B), indicating the specificity of the DNA-protein interaction. Expression analysis showed that *BrOPR3* is significantly upregulated by exogenous MeJA treatment (Figure S1).

### 2.5. BrTCP7 Activates the Transcription of BrOPR3 and BrRCCR

**Figure 5:**
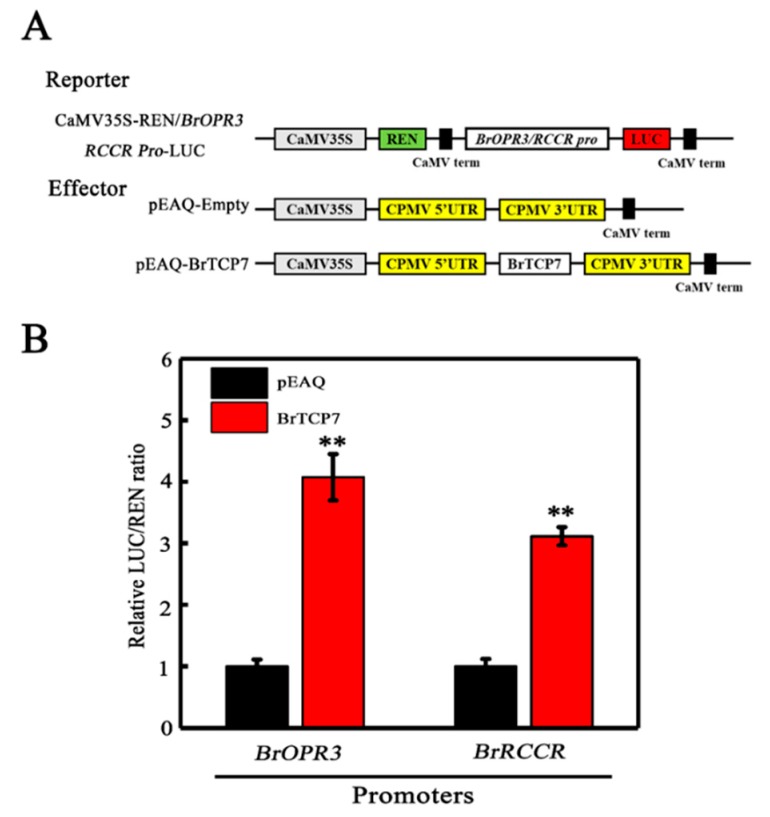
BrTCP7 enhances *BrOPR3* and *BrRCCR* transcriptions by transient transcription dual-luciferase assay in *Nicotiana benthamiana* leaves. (**A**) Diagrams of the reporter and effector vectors. (**B**) BrTCP7 activates *BrOPR3* and *BrRCCR* promoters. Data are means ± S.D. of six independent biological replicates. Asterisks indicate significant differences by student’s *t*-test (** *p* < 0.01).

After deciphering *BrOPR3* and *BrRCCR* as the potential downstream targets of BrTCP7, and establishing BrTCP7 as a transcriptional activator, we hypothesized that BrTCP7 could act as a direct transcriptional activator of *BrOPR3* and *BrRCCR*. To investigate this hypothesis, *Nicotiana benthamiana* leaves were co-transformed with transient over-expression effector construct containing 35S-BrTCP7, and a dual-luciferase reporter construct carrying *BrOPR3* or *BrRCCR* promoter fused to LUC (Figure 5A). As illustrated in Figure 5B, expression of *BrTCP7* resulted in 3.07- and 2.11-fold increases in *LUC* expression driven by *BrOPR3* and *BrRCCR*, respectively, compared with the vector control, providing evidence for a positive trans-activation activity of BrTCP7. Collectively, BrTCP7 binds directly to the promoters of *BrOPR3* and *BrRCCR* and activates their transcriptions, rendering them direct targets of BrTCP7 transcriptional regulation. Further experiments, such as chromatin immunoprecipitation (ChIP) assay or transient over-expression of *BrTCP7* in *Brassica* protoplasts, are needed to confirm the binding of BrTCP7 to *BrOPR3* and *BrRCCR* in vivo.

Similar to BrTCP7, Arabidopsis class II CINCINNATA (CIN)/TCP TFs, such as TCP4, positively influence JA biosynthesis by directly activating the transcription of a JA biosynthetic gene *LOX2* [32]. Conversely, the class I TCP members TCP9 and TCP20 are negative regulators of leaf senescence via repressing *LOX2* expression [32]. These findings highlight the complexity of TCPs in modulating JA-promoted leaf senescence. Auxin usually promotes leaf senescence and functions downstream of class I and CIN TCPs by regulating the same auxin-responsible TFs, indicating the possible involvement of TCPs in auxin-modulated leaf senescence [39,40]. A class I TCP TF GhTCP19 from gladiolus was reported to positively regulate corm dormancy release by suppressing the transcription of an ABA biosynthetic gene *GhNCED* while activating the transcription of cytokinin biosynthetic gene *GhIPT* and signal transduction gene *GhARR* [29]. As ABA and cytokinins have been implicated to accelerate and delay leaf senescence in Chinese flowering cabbage, respectively [4,37], investigating whether BrTCP7 or other TCP members like GhTCP19 have regulatory roles in ABA-, cytokinins-, and auxin-mediated leaf senescence will be required.

Transcriptional activity of TFs on their targets is finely controlled by many factors, such as homo-/hetero-dimerization, post-transcriptional/translational modification, and protein–protein interactions [41]. For example, miR319a/JAGGED targets class II TCPs and subsequently represses *LOX2* expression, which attenuates JA-induced senescence [26,30]. A study in bananas showed that MaTCP20 is associated with MaTCP5 or MaTCP19 to form complexes, which influence the regulation of cell wall-modifying genes during fruit ripening [42]. An ERF TF BrERF72 was found to be associated with MeJA-promoted leaf senescence in Chinese flowering cabbage by enhancing JA accumulation through upregulating the expression of three JA biosynthetic genes: *BrLOX4*, *BrAOC3*, and *BrOPR3* [5]. Thus, elucidating whether BrTCP7 and BrERF72 can form a complex to affect the expression of JA biosynthetic genes, as well as identifying other interaction proteins and regulatory factors, will provide more detailed information about the BrTCP7-mediated regulation of gene networks operating MeJA-promoted leaf senescence of Chinese flowering cabbage. Targeted transgenic research is required to unravel the biological function of BrTCP7 in regulating MeJA-promoted leaf senescence in Chinese flowering cabbage.

## 3. Materials and Methods

### 3.1. Plant Materials, MeJA Treatment, and Growth Conditions

Chinese flowering cabbages (*Brassica rapa* var. parachinensis) grown in a local commercial vegetable plantation near Guangzhou, Southern China, were harvested after 40 days of growth, and were immediately transported to laboratory under low temperature. Uniform size cabbages without appearance defects were selected and randomly divided into two groups for control and MeJA treatment. MeJA (100 μM) and distilled water (control treatment) were foliar-sprayed onto cabbages as described previously [5]. Subsequently, both control and MeJA-treated cabbages were stored at 15 °C in incubators with a 16-h light/8-h dark cycle for 5 days. On days 0, 1, 3, and 5 of storage, the third leaves from the bottom of 10 cabbages were collected for physiological and molecular analysis. All samples were immediately frozen in liquid nitrogen and stored at −80 °C for further assays.

Tobacco (*Nicotiana benthamiana*) plants were planted in a growth chamber set at 22 °C and a 16-h photoperiod. Leaves from four-week-old tobacco plants were used for *Agrobacterium-tumefaciens*-mediated transient expression assays.

### 3.2. Physiological Measurements of Leaf Senescence

Leaf total chlorophyll content and photochemical efficiency (Fv/Fm) are physiological parameters commonly used as indicators of leaf senescence. Total chlorophyll content was measured by extracting approximately 0.1 g of leaves in 10 mL in 80% acetone in the dark for 24 h and measuring the absorbance of extracts at 663 nm and 645 nm using a spectrophotometer as described earlier [5,6]. *Fv*/*Fm* was determined non-invasively after leaves were adapted to dark conditions for 30 min. A chlorophyll fluorometer (Imaging-PAM-M series, Heinz Walz GmbH, Effeltrich, Germany) equipped with a charge-coupled device (CCD) camera to capture high-resolution digital images of the emitted fluorescence from the dark-adapted leaves.

### 3.3. RNA Extraction, Gene Cloning, and Bioinformatic Analysis

A Quick RNA Isolation Kit (Huayueyang, Beijing, China) was used to extract total RNA from cabbage leaves. RNA was then treated with DNase I to remove contaminating DNA and reversely transcribed to cDNA using the reverse transcriptase M-MLV (TaKaRa, Shiga, Japan) using 1 μg RNA template, following the manufacturer’s protocol. The full-length BrTCP7 gene was isolated from our transcriptome database. Theoretical isoelectric points (*p*I) and mass values were calculated using the Compute *p*I/Mw tool (http://web.expasy.org/compute_pi/). Sequence alignment and phylogenetic analysis of TCP proteins were conducted with the CLUSTALW program (version 1.83, The Conway Institute of Biomolecular and Biomedical Research, University College Dublin, Dublin, Ireland). A phylogenetic tree was created with the MEGA5.0 program (Institute of Molecular Evolutionary Genetics, The Pennsylvania State University, PA, USA), using the maximum likelihood inference method.

### 3.4. Gene Expression via qRT-PCR

cDNA from each sample were quantified by quantitative real-time PCR (qRT-PCR) assays. qRT-PCR was performed using the step one plus a CFX96 Touch™ real-time PCR detection system (Bio-Rad, Hercules, CA, USA) and GoTaq qPCR master mix kit (Promega, Madison, WI, USA). The expression levels of target genes were normalized according to the cycle threshold (*C*t) value using *BrActin1* [43] as the reference gene.

### 3.5. Subcellular Localization Assay

The BrTCP7 coding sequence (CDS) fragment without the stop codon was amplified and inserted into the pEAQ-GFP vector [44] to produce the fusion protein BrTCP7-GFP. Then BrTCP7-GFP and the control pEAQ-GFP constructs were transformed to *A. tumefaciens* strain EHA105. Overnight cultures of *Agrobacteria* were collected by centrifugation, resuspended in MES buffer to 0.8–1.0 OD_600_, incubated at room temperature for 2 h before infiltration. *Agrobacteria* suspension collected in a 1-mL syringe (without the metal needle) was carefully press-infiltrated manually onto healthy leaves of 4-week-old *Nicotiana benthamiana* as described previously [45]. Two days after infiltration, the GFP fluorescent signals in the epidermal cells of leaves were directly observed and images were captured by using a Zeiss fluorescence microscope (Carl Zeiss AG, Oberkochen, Germany).

### 3.6. Recombinant Protein Induction, Purification, and EMSA Assay

For protein expression and purification, the BrTCP7 coding sequence was recombined into the vector pGEX-4T-1 and transferred to *Escherichia coli* strain Transetta (DE3). When the transformed DE3 cell density reached 0.6 (OD_600_), protein expression in 1-L cultures maintained at 37 °C was induced by the addition of 0.3 mM isopropyl thio-β-D-galactoside during a 3 h course. Cells were harvested by centrifugation. The cell pellet was resuspended in phosphate-buffered saline and incubated on ice for 30 min. Following sonication, the lysate was cleared by centrifugation. Glutathione-Superflow Resin (Clontech, Mountain View, CA, USA) was used for purification of GST-tagged protein by gravity-flow chromatography according to the manufacturer’s protocol, followed by SDS-PAGE and Coomassie Brilliant Blue staining to confirm protein size and purity.

Electrophoretic mobility shift assay (EMSA) was performed as described previously [46]. The synthetic nucleotides (~60 bp) derived from the 5′ UTR of *BrOPR3* and *BrRCCR* oligonucleotides, which contain the consensus binding site (GGNCCCAC) of TCPs, were biotin-labeled at the 5′ end. The purified recombinant BrTCP7 protein (1 μg) was incubated with biotin-labeled probe (2 × 10^−6^ μmol) in binding buffer for 25 min at 30 °C. Competitions were conducted by adding excess amount (250-fold) of cold probe (unlabeled DNA fragment) or mutant probe. The reaction mixture was electrophoresed on a 6% native polyacrylamide gels, and then transferred onto a positively charged nylon membrane, followed by cross-linking though illumination under an ultraviolet lamp. The signals from the labeled DNA were detected by using the LightShift chemiluminescent EMSA kit (Thermo Scientific, Rockford, IL, USA) in a ChemiDoc™ MP Imaging System (Bio-Rad, Hercules, CA, USA).

### 3.7. Transient Transcription Dual-Luciferase (Dual-LUC) Assays

To investigate the transcriptional ability of BrTCP7 in vivo, its full length was inserted into pBD [45] to construct pBD-BrTCP7 as an effector. The positive control (pBD-VP16) was constructed by fusing VP16, a herpes simplex virus-encoded transcriptional activator, to pBD. pBD itself was used as a negative control. The GAL4 plasmid with the *firefly luciferase* (*LUC*) gene was used as a reporter [45], and the *renilla luciferase* (*REN*) gene in the same plasmid was used as an internal control.

To determine the activation of *BrOPR3* and *BrRCCR* by BrTCP7, the promoter fragments of *BrOPR3* and *BrRCCR* were amplified and cloned into the transient dual luciferase expression vector pGreenII 0800-LUC [47] as reporter constructs. To generate the 35S::BrTCP7 effector construct, the BrTCP7 coding sequence was amplified by PCR and inserted into pEAQ vector [44]. The empty vector was included as a control.

Transient transcription dual-LUC assays were performed using *Nicotiana benthamiana* plants as described [46]. The reporter and effector constructs mentioned above were co-infiltrated into tobacco leaves. After 2 days of infiltration, the luciferase activity of tobacco leaf extract was quantified by a Luminoskan Ascent Microplate Luminometer (Thermo Fisher Scientific, Rockford, IL, USA), using commercial dual-luciferase reporter assay kit according to the manufacturer’s instruction (Promega, Madison, WI, USA). The trans-activation ability of BrTCP7 was indicated by the LUC/REN ratio.

### 3.8. Statistical Analysis

Data are represented as means ± SD of three or six biological replicates. Statistical differences of two treatments were examined using the Student’s *t*-test. Data are considered significant as follows: * *p* < 0.05, ** *p* < 0.01

### 3.9. Primers

All primers used in this research are listed in Table S1.

## 4. Conclusions

In summary, exogenous MeJA treatment promotes leaf senescence of Chinese flowering cabbage. A class I TCP member BrTCP7, which is MeJA-upregulated and acts as a nuclear-localized transcriptional activator, was identified. BrTCP7 targets the promoters of a JA biosynthetic gene *BrOPR3* and a *CCG* gene *BrRCCR*, leading to their transcriptional activation. These findings expand our understanding of TCP TFs’ functions and shed light on the transcriptional mechanism operating JA-mediated leaf senescence, and also the molecular mechanism(s) involved in maintaining postharvest quality of an important leafy vegetable, Chinese flowering cabbage.

## Supplementary Materials

Supplementary materials can be found at https://www.mdpi.com/1422-0067/20/16/3963/s1.
