# Supplementary material for: BrTCP7 Transcription Factor Is Associated with MeJA-Promoted Leaf Senescence by Activating the Expression of BrOPR3 and BrRCCR
Source: Int J Mol Sci. 2019 Aug 14;20(16):3963. doi: 10.3390/ijms20163963 (PMC6719003; doi:10.3390/ijms20163963)
Supplement: Supplementary file 1 [file ijms-20-03963-s001.pdf]

Supplementary materials

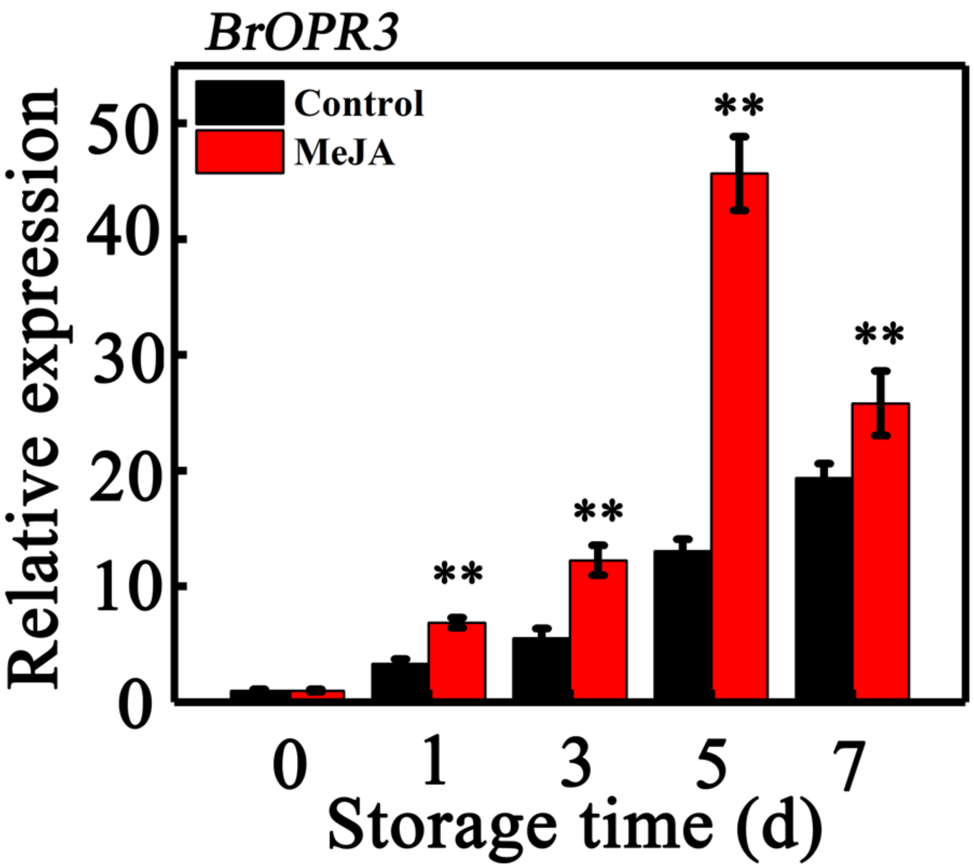

Figure S1. Relative expression of *BrOPR3* in control and MeJA-treated cabbage leaves during senescence. Each value represents the mean ± S.E. of three biological replicates. Asterisks indicate a significant difference in MeJA-treated leaves compared with control leaves (Student's *t*-test: $^{**}P < 0.01$).

Table S1. List of primers used in this study.

Assay	primer sequence			Restriction Site
		Forward primer(5'-3')	Reverse primer(5'-3')	
**RT-qPCR**	***BrActin1***	CGCTTAACCCGAAAGCTAAC	TACGCCCACTAGCGTAAAG	
	BrTCP7	CAGCAAAGTCGACGGAAGAG	AGAGAGTGCAGTTCGTGGAA	
	BrOPR3	CGGACCAGACCGTGTTGGCATTA	TCATAGGCACAAGCAAGTGAGGACA	
	BrRCCR	AGACTCACCGTTCAAGCTCA	AAGGAGTTGAGAGTGGAGGC	
	BrNYC1	CGGAGACGGTGGCTAGAACG	TGCTTCTCCTGAGCCACGAC	
	BrPPH1	TATCTGATGCGCGGGTGGAT	TTCCCGACCAATGCTGGACT	
	BrPAO1	CAGCTTCAGCGACACTCACC	TCGCCGTGCTCTTCTTCGAT	
	BrSGR1	GTTGGGGTCCGCTTTGGGAA	AATCGAGCTAACCTGCGGGA	
	BrSGR2	GCAGCACAACCACGATGGAA	CGATGAGACCACGGGATCGT	
	BrSAG12	CACTGGCGGCTTAACCACTGAA	GAAGATTGGCTGTATCCTACGGC	
	BrSAG19	GCAAGCGAGCGTTGGTAAAGGT	GGGTTGATTCTTCCACTCCCTTC	
**Subcellular localization**	***BrTCP7-GFP-For***	ATCTAGAGCAGTCGACGGTACCATGTCTAATAACGACGGAGTTATGA		***AgeI***
	BrTCP7-GFP-Rev	CTCCTCGCCCTTGCTCACCATACGCTGGTCATCCTCTCTCC		***AgeI***
EMSA	***pGEX-4T-1-BrTCP7-For***	GGTTCCGCGTGGATCC ATGTCTAATAACGACGGAGTTATGA		***BamHI***
	pGEX-4T-1-BrTCP7-Rev	AGTCACGATGCGGCCGC TCAACGCTGGTCATCCTCTC		***NotI***
	EMSA -BrRCCRpro-For	ATGAGAAGAATTTCGTGGTGGGTGGGTCCCATATGACGGAGATAAAGAAC		
	EMSA -BrRCCRpro-Rev	GTTCTTTATCTCCGTCATATGGGACCCACCCACCACGAAATTCTTCTCAT		
	EMSA –BrOPR3pro-For	GCTAGACAGATACATACCTCCGGTCCCACCTGAACCAATCATAGCCTGGC		
	EMSA –BrOPR3pro-Rev	GCCAGGCTATGATTGGTTCAGGTGGGACCGGAGGTATGTATCTGTCTAGC		
**Dual LUC assay**	***pBD-BrTCP7-For***	TCGCCGACCGGTAGGCCTATGTCTAATAACGACGGAGTTATGA		***Stu I***
	pBD-BrTCP7-Rev	AACCAGAGTTAAAGGCCTTCAACGCTGGTCATCCTCTC		***Stu I***
	pEAQ-BrTCP7-For	CAAATTCGCGACCGGT ATGTCTAATAACGACGGAGTTATGA		***AgeI***
	pEAQ-BrTCP7-Rev	AGTTAAAGGCCTCGAG TCAACGCTGGTCATCCTCTC		***XhoI***
	0800-BrRCCR-For	TATAGGGCGAATTGGGTACCAACGGTACCGGACAAGTGG		***KpnI***
	0800-BrRCCR-Rev	TTGGCGTCTTCCATGGCTTTTATTATATGTGGCTGTG		***NcoI***
	0800-BrOPR3-For	TATAGGGCGAATTGGTGGGCTTGTATGGTTTGGGCC		***KpnI***
	0800-BrOPR3-Rev	TTGGCGTCTTCCATGGGAATTTCTGTGATTCTTTTTCTCTCA		***NcoI***

Text S1. Promoter nucleotide sequences of *BrOPR3* and *BrRCCR*. GTGGTCCC is marked by red and boxed. Translation start site (ATG) was shown in yellow box.

BrOPR3 **(**NW_008711256.1) promoter
TTTGGGCTTGTATGGTTTGGGCCGCGGGCCGTTTATAGCATTTAGACTGTAGCCGGTTTGGCTTATGGGCTTCGTGCCT
TTTTGTTTATGCGTTTGGGCTTCAGACTATGTAATTAGGCTTGGCCCGGTATTATTATTAATCTAAATAAAATCTTGACG
GAAAAAAAAAAAAAAAGAATGTCACACATGTACAATATGTACAAAGGATCTTTTTTGAATAAGCAAGAAACAGCTAG
ACAGATACATACCTCCGGTCCCACCTGAACCAATCATAGCCTGGCGGCTAAGTCTTACAGTTGAATTGTTCATGGGTT
GTCCATATGGTGATGTGAACCATACACATTACACTAAATACTGATAAAAAAGGATACCAACTCTTCTATTCGACTTAT
GTTTTTCGGTCTTGAATTTCTAAATCAGTTACTCTAAATCAACAACTTATGTTTTTCAAATCTGAACTTTTATGTTACTC
TAAATCTTCTATTCGAGCATTCATTTCAGTCTCAAATCCCGTTATGTATGGTGCTATCTTTCTTTCCTTTTTACTCGAAC
ACCTTAACCTTTTGTTCTGTCGGTGGTCAAAGTTGGACAAAGTCAATCCAATTATTTTCTTTTTAATTATTAAAATTATA
TGAGGCCCATTAAAGTTTTGTTCTGTAATTATTCTCTAAAAGTCGAATCTCAACTGTAGGACGGAGTAAGAAAGAAAC
GAGCATTATTGTGGTTAAGTTCAAAGTGGTGGTTACTCTCTAATGTCGTCAATGCATACGTCATGTAAATGCAAGTGG
ATGGGGTCCACGGATGCATGCGTCCTCAATGACGTCCACGTCCAAAGAAAAAACTTACTTTCCTAATATGTATATCTA
ACCAAATGTGTATATTTGAACAAAAAAAAAAAAACCAAATGTGTATATATATATGTCGCAGATATAACCATCTTCGTCC
ACAACTCACCAAAAACTTGAAAGTGTTTTGAGAGAAAAAGAATCACAGAAATTCATG

BrRCCR (XM_009140630.2) promoter
AACGGTACCGGACAAGTGGGAGTTCGCTAACGACAACTTCCGGAGAGGACAAGAGGAACTGCTGTCGGAGATACGGC
GGCGCAAGGCGGTGATCGCCGCGGCAGGGAAATGCGTAGTCGTTGGTTCGCCGTCGGAGTCAAACTCGGCGGGTGAC
GATCACGGTTCTAGCTCCACGTCATCGCCGGGGTCGAAGCACCCGGGGTCGGTGGAGAACATGGTTGCTGACTTATCC
GGGGAGAACGAGAAGCTGAAAAGAGAGAACAGCAGTTTGAGCTCGGAGCTCGCGGCGGCGAAGAGGCAGCGCGACG
AGCTGGTGGCGTTCTTGACCGAGCAGATGAAAGTGGGACCGGAGCAGATCGATCAGATGATCAAAGGAGGAGGGAA
GAAACTCAAACCGGCGGTGGAGGAGGAAGAGAGCGACTGCGAAGGCTGCGGCGGAGACAACGGTGGAGCCGCCGTG
GAGGGAGAGAAGGGGGTGGTAGGTGAAGGTTTGAAACTGTTTGGGGTATGGGTGAAAGGAGAGAGAAAGAAGAGGG
GCCGGGATGAGAAGAATTTCGTGGTGGGTGGGTCCCATATGACGGAGATAAAGAACGTGGACTTTCACGCGCCGTTG
TGGAAGAGCAGCAAAGTCTGCAACTGATAAAGACTTCTAGATACTTGTTCAAACAAACGTGTGACACGTCATTGACGA
AGAGAAGAAAAAAGAGAAAGCTGCAAGTTTTAATGATTTTTAATAATTTTCCGTAAGGGAGATTAGTGCTTTATTTTT
ATGTTAAAATTGGATTTTCATGTATTATATACTTTATTAGTGTATTAAGTTTGAAAAAAAATAAGACAAAACGTTTCGG
ATTTTTACTATTGGGTGTATCTACTTTTCTCTTAAAAACTTCTAGCTAATGTAAAAGCACGGGATTAGATTTGGTCCAG
GGTCACGTTGAAACTTCAAAGAAGAGGATTAGTAATTGTCAGAACTTGACTACTTCTCTTGCAATTAGTAGGTAGTTTC
CGTGTTTATACGTAACCCGTCGAATATTTTCATACTAATTGTCAGAAAAGTCGACAACAAAGACAACATTGTATCGAA
GGAAATGTAATTGGAGAAATACCTAACTCTTACATAACACAAGGCATCAAAAATTCCAACAAAAATAATACTACGAG
ATTAAATAAAAAATTATGGTAATGAGAAAAATATTTCAACTATTAGCTCTACGACAACTAAGGTTATAATTAAACTAA
ATTCGCTAAGGATAGATATATTCTAAAATTTTGTCGCTTAATGAATCCTAACCCATTTTCTTACATAATAAACATATTTT
CAGACGGAATATTTGAATAGTATATTCGTTGATGCCCAATTAAGTCGCTCTTGAGTCTTGCCAAGACTAGAACATCATT
TTAGTAGAAAATCAATACCCACTAACTCTCAAGCATTAGAAAACATAGTATCTTTCTATGTTTAGCTTTATGTAATTCA
CACACGCATATTTTTAAACGGGTTCGGTTTAGTGTATTTTGACGTCTAAAATATAATTTTTGTTTATAGATTATTATATT
AATTTTTATAATAAATTTTGATGTTTAATATATGATATTTTCGTGATTTTTTTTTTTACTTTAAAACAACACGCTTTTTAA
TATATTTTTTCTTTATTATGTAATTAGTTTTTGTAGATGACAATTGGCAAAATATCTTTTTATACTTTGAGAAAGTGTAA
ATAATTCTAAAGAAGTGTGTAATATAAAGAAGAAAGCTGATATTTACTCACAGCCACATATAATAAAAGATG
